# Naive and Memory B Cell BCR Repertoires in Individuals Immunized with an Inactivated SARS-CoV-2 Vaccine

**DOI:** 10.3390/vaccines13040393

**Published:** 2025-04-08

**Authors:** Renato Kaylan Alves de Oliveira França, Pedro Henrique Aragão Barros, Jacyelle Medeiros Silva, Hitallo Guilherme Costa Fontinele, Andrea Queiroz Maranhão, Marcelo de Macedo Brigido

**Affiliations:** 1 Department of Cellular Biology, Institute of Biological Science, University of Brasília, Brasilia 70910-900, DF, Brazil; renatokaylan@gmail.com (R.K.A.d.O.F.); pedroaragaobarros@gmail.com (P.H.A.B.); hitalloguilherme@hotmail.com (H.G.C.F.); andreaqm@unb.br (A.Q.M.); 2 Molecular Pathology Post-Graduation Program, University of Brasília, Brasilia 70910-900, DF, Brazil; 3 Molecular Biology Post-Graduation Program, University of Brasília, Brasilia 70910-900, DF, Brazil; 4 III-Immunology Investigation Institute, National Institute of Science and Technology (iii-INCT), Brasilia 70067-900, DF, Brazil

**Keywords:** BCR, memory B cell, VH repertoire, CoronaVac, SARS-CoV-2

## Abstract

Background: The COVID-19 pandemic has spurred a global race for a preventive vaccine, with a few becoming available just one year after describing this novel coronavirus disease. Among these are inactivated virus vaccines like CoronaVac (Sinovac Biotech), which are used in several countries to reduce the pandemic’s effects. However, its use was associated with low protection, particularly against novel virus variants that quickly appeared in the following months. Vaccines play a crucial role in activating the immune system to combat infections, with Memory B-cells being a key part of this mechanism, eliciting protective neutralizing antibodies. This work focused on studying B-cell memory repertoire after two consecutive doses of CoronaVac. Methodology: Memory B-cells were isolated from five CoronaVac vaccinated and five pre-pandemic individuals and subsequently stimulated in vitro before high-throughput Illumina sequencing of the Heavy Chain Variable repertoire. Results: We observed a shift in the VH repertoire with increased HCDR3 length and enrichment of IGVH 3-23, 3-30, 3-7, 3-72, and 3-74 for IgA BCRs and IGHV 4-39 and 4-59 for IgG BCRs. A high expansion of IgA-specific clonal populations was observed in vaccinated individuals relative to pre-pandemic controls, accompanied by shared IgA variable heavy chain (VH) sequences among memory B cells across different vaccine recipients of IgA clones was also observed in vaccinated individuals compared to pre-pandemic controls, with several IgA VH sharing between memory B cells from different vaccines. Moreover, a high convergence was observed among vaccinees and SARS-CoV-2 neutralizing antibody sequences found in the CoV-abDab database. Conclusion: These data show the ability of CoronaVac to elicit antibodies with characteristics similar to those previously identified as neutralizing antibodies, supporting its protective efficacy. Furthermore, this analysis of the immunological repertoire in the context of viral infections reinforces the importance of immunization in generating convergent antibodies for the antiviral response.

## 1. Introduction

The COVID-19 pandemic has mobilized a tremendous global effort to develop antiviral therapies with novel vaccines, new antiviral small molecules, and therapeutic antibodies [1]. Infection by SARS-CoV-2, the etiological agent of COVID-19, leads to severe respiratory syndrome, vascular inflammation, and neurological complications with life-long consequences [2]. The pandemic is now under control partly due to several vaccines, including novel formats like mRNA-based vaccines.

One of the earliest vaccines made available was CoronaVac, developed by Sinovac Biotech. This vaccine uses an inactivated whole virus based on the original Wuhan variant [3]. CoronaVac was applied in China, Brazil, and Türkiye, among other countries, and despite its traditional manufacturing, it is protective and can protect against severe COVID-19. However, a reduction in the protection generated by CoronaVac was observed throughout the pandemic and the evolution of the virus [4,5].

The immunoprotective response against SARS-CoV-2 involves the activation of specific B cell clones and the production of neutralizing antibodies against different viral antigens [6]. IgG and IgA memory responses have been identified as essential to control the infection, especially those derived from clones harboring the IGHV1, IGHV3, and IGHV4 variable gene segment families [7,8]. It has been shown that IgA dominates the first neutralizing response and that mucosal IgA antibodies have greater neutralizing capacity than serum IgG and IgA [9]. In addition to classical memory B cells, naive IgM antibodies or antibodies with low somatic hypermutation have significant neutralizing capacities against SARS-CoV-2. These anti-SARS-CoV-2 IgM and near-germinal IgG have been associated with the cross-response against other coronaviruses. They may be related to the humoral memory derived from previous contacts with recurrent coronaviruses, such as HKU1, OC4, and 229E, or previous pandemics with SARS-CoV-1 and MERS-CoV [10,11,12].

The study of memory B cells (MBC) is hampered by the low percentage of this cell population in peripheral blood [13]. Cell sorting or ELISPOT techniques can overcome this obstacle. However, these methodologies may result in the depletion of clonal populations critical to the antiviral immune response under investigation due to factors such as cell viability after separation, differences in expression or secretion of markers, and differences between the binding capacity of the BCR/antibody and its respective neutralizing property [14,15]. In addition, rare B cell clones, such as atypical memory populations, which, although low in number, present high neutralization potential, may not be recovered [16]. Approaches of immune cell activation and expansion with polyclonal stimulation factors, like cytokines and TLR agonists, may be an alternative for studying the antibody repertoire in the context of infections [17]. We have previously demonstrated that ex vivo culture of peripheral blood mononuclear cells (PBMCs) with the stimulatory agents interleukin-2 (IL-2) and Toll-like receptor 7/8 (TLR7/8) agonist R848 facilitates the expansion and isolation of virus-specific memory B cells. This approach is practical even in PBMCs from individuals with antigenic exposure occurring up to two years prior, with further enhancement observed when PBMCs are pulsed with the specific viral antigen. This method significantly enriches circulating memory B cell populations in PBMC culture, including rare clonal subsets, and is therefore highly suitable for immunoglobulin repertoire studies [18].

CoronaVac, an inactivated whole-virion vaccine, has not been extensively characterized in terms of its impact on the antibody repertoire, particularly in comparison to other approved SARS-CoV-2 vaccines, such as mRNA and viral vector-based vaccines, which utilize only the spike protein antigen [19,20,21]. Exploring the gap regarding the immunoglobulin repertoire in the context of immunization with CoronaVac can contribute to the study of this vaccine’s effects and compare these effects with humoral immunity in response to natural infection and to other SARS-CoV-2 vaccines. To explore the dynamics of the antibody response repertoire after the CoronaVac vaccination, we applied the previously established B cell expansion protocol, followed by high-throughput sequencing. Comparing the CoronaVac vaccinated with pre-COVID pandemic control immune repertoires, we observed repertoire changes marked by the appearance of antibodies correlated to known SARS-CoV-2 reactive and neutralizing ones derived from infected and vaccinated individuals.

## 2. Materials and Methods

### 2.1. Cohort of Individuals

Five individuals vaccinated with two doses of the CoronaVac vaccine (Sinovac, Beijing, China) were selected to study the B-cell immune response to this vaccination. The individuals were vaccinated in 2021, with an interval between doses of 28 days. Each dose of the vaccine corresponded to 0.5 mL of injectable suspension containing 600 SU of the inactivated SARS-CoV-2 virus antigen (original Wuhan lineage, entire viral particle). All selected individuals signed a consent form and answered a clinical and technical information questionnaire. The exclusion criteria were being under 18 years old, over 59 years old, and having an immunodeficiency. Peripheral blood samples (80 mL) were collected from individuals 30 days after the second dose and were used to isolate PBMC (peripheral blood mononuclear cells) and obtain serum.

This research project was approved by the Research Ethics Committee of the Medical School of the University of Brasília under CAAE opinion 46661221.7.0000.5558 from 8 October 2021. All experiments were conducted following the human research ethics regulations of the National Research Ethics Committee (Brazil), and all participants signed an Informed Consent Statement.

### 2.2. B Cell Expansion and Isolation Protocol

PBMC were cultured for seven days for cell expansion in a RPMI medium with 10% FBS, 1× Antibiotic-Antimyotic solution (Thermo Fisher Scientific, Roskilde, Denmark, cat: 15240062), human IL-2 at 5 ηg/mL (Sigma-Aldrich, St. Louis, MO, USA, cat: SRP6170) and TLR-7/8 agonist at 1 µg/mL, R848 (Resiquimod, Sigma-Aldrich, cat: SML0196). The cells were incubated at 37 °C and 5% CO_2_ for seven days. The supernatant was collected during cultivation on the third, fifth, and seventh day for ELISA analysis.

After stimulating the culture for seven days, memory B cells and naive B cells were purified from PBMC using the human Memory B Cell Isolation Kit (Miltenyi Biotec, Bergisch Gladbach, Germany, cat: 130093546). Briefly, 6 × 10^7^ cells were centrifuged at 1800 rpm for 8 min and resuspended with 240 µL of ice-cold MACS buffer (PBS with 0.5% FBS). Then, 60 µL of Memory B-cell biotin antibody cocktail (a cocktail of biotinylated antibodies specific to non-B-cell markers; antibodies anti-CD2, CD14, CD16, CD36, CD43, and CD234a) were added and incubated at 4 °C for 10 min. Then, 180 µL of ice-cold MASC buffer and 120 µL of anti-biotin microbeads (magnetic microbeads conjugated to an anti-biotin monoclonal antibody) were added, and incubated at 4 °C for 15 min. After incubation, 6 mL of ice-cold MASC buffer was added, and the mixture was centrifuged at 1800 rpm for 8 min. The pellet was resuspended with 1 mL of MACS buffer and applied to the LD magnetic separation column (Miltenyi, cat: 130042901, capacity of 10^8^ labeled cells). In this first negative selection, other cells (T cells, NK cells, monocytes, dendritic cells, granulocytes, platelets, and erythrocytes) are labeled and retained, leaving the total, unlabeled B cells, which freely traverse the column and are rescued in RPMI medium.

Total B-cells were centrifuged at 1800 rpm for 8 min. The supernatant was discarded, and the pellet was resuspended with 10 µL of ice-cold MACS buffer. Ten µL of CD27 microbeads (magnetic microbeads conjugated to an anti-CD27 monoclonal antibody, an anti-memory cell marker) were added and incubated at 4 °C for 15 min. Then, 6 mL of ice-cold MASC buffer was added, and the mixture was centrifuged at 1800 rpm for 8 min. The pellet was resuspended with 500 µL of MACS buffer and applied to the magnetic MS separation column (Miltenyi, cat: 130042201, capacity of 107 labeled cells). In this second positive selection, memory B cells were labeled (anti-CD27) and retained in the column, while naive B cells freely traversed the column and were rescued in the RPMI medium. The memory B cells were then collected after removing the column from the magnetic base.

### 2.3. Analysis of Flow Cytometry

Before and after the culture, the cells were stained with antibody cocktail for human memory B cells and naive B cells: anti-CD19 APC (BD, Franklin Lakes, NJ, USA, cat: 555415), anti-CD27 PE (BD, cat: 560985), anti-IgG FITC (Thermo Fisher, cat: A18818), anti-IgD FITC (Thermo Fisher, cat: 11-9868-42) and anti-IgA FITC (Thermo Fisher, cat: H14001).

At the end of seven days of culture, cell death/viability analysis was performed with the kit Annexin-APC/PI (eBioscience™, San Diego, CA, USA, cat: 178007).

In analyzing memory B cells and their subpopulations for each sample, an average of 100,000 events were captured within the lymphocyte gate. The percentage of the cells was calculated according to the number of total PBMC (ungated events). The absolute cell count was calculated concerning the total number of acquired (ungated) events corrected concerning the number of viable cells, considering Annexin-V/PI staining. The flow cytometry data were analyzed in the FlowJo Software version 8.0.

### 2.4. Cell Proliferation Analysis

Cell proliferation was analyzed using a CFSE assay. For this, each 1 × 10^6^ cells were stained with 100 µL CFSE 10 µM (Thermo Fisher, cat: C34554) at 37 °C for 10 min, under protection from light. After, the free reagent was inactivated with FBS 1:2. 1 × 10^6^ stained cells were plated in 96-well plates, with the proportion of 1 × 10^6^ cells per well, and were cultivated in 1 mL of RPMI with 10% SFB, 5 ηg/mL IL-2 and 1 µg/mL R848 for seven days. A control condition was prepared, and the cells were cultivated in a medium with only FBS and DMSO (1%), which were used to dilute the R848. After cultivation, the cells were analyzed by flow cytometry with additional anti-CD19 APC and anti-CD27 PE staining.

### 2.5. ELISA for Quantification of Total Immunoglobulins

ELISA quantified human IgM, IgG, and IgA in the supernatants of the PBMC expansion culture on days 3, 5, and 7 of cultivation. For this, 100 µL of human IgG/IgA/IgM anti-H+L antibody (Thermo Fisher, Waltham, MA, USA, cat: 31128), diluted 1:1000 in PBS, were adsorbed in a NUNC MaxiSorp flat-bottom 96-well ELISA plate (Thermo Fisher Scientific, cat: 44-2404-21) at 4 °C, overnight. The following day, the wells were washed three times with 200 µL of PBST (PBS with 0.1% Tween 20) and blocked with 200 µL of 1% Bovine Serum Albumin (BSA) (Sigma-Aldrich, cat: A2153) in PBST, at 37 °C for 1 h. The wells were washed, as before, and 100 µL of the diluted supernatants were added. The supernatants from the third, fifth, and seventh days were diluted 1:2, 1:20, and 1:200 in PBS, respectively. Standard curves for each immunoglobulin were added with seven 2-fold serial dilutions 330, 167, 83, 42, 21, 10, and 5 ηg/mL). The plate with the supernatants and curves was incubated at 37 °C for 1 h. Then, the wells were washed, and 100 µL of anti-human IgG (Thermo Fisher, cat: A18820), IGA (Thermo Fisher, cat: 31314) or IGM (Thermo Fisher, cat: A3437) conjugated to alkaline phosphatase (AP), all diluted 1:2500 in PBST, were added, and the plate was incubated at 37 °C for 1 h.

After washing the plate with PBST, captured IgMs, IgGs, and IgAs were detected with 100 µL of 1 mg/mL pNPP substrate (Thermo Fisher Scientific, cat: 34045). The plate was incubated with the pNPP solution at room temperature for 30 min, and reading was performed with a 405 nm filter on the SpectraMax M2e spectrophotometer (Molecular Devices, San Jose, CA, USA).

### 2.6. Binding of Antibodies to SARS-CoV-2

The presence of IgM, IgG, and IgA, specific to SARS-CoV-2, in the sera from individuals and in the culture, supernatants were measured by ELISA. The sera were collected 30 days after the second dose of CoronaVac, and the supernatants were collected on the third, fifth, and seventh days of PBMC culture. A NUNC MaxiSorp 96-well plate (Thermo Fisher Scientific, cat: 44-2404-21) was coated with 100 µL of 1 × 10^3^ PFU SARS-CoV-2 (Wuhan lineage, kindly provided by Dr. Pedro Mendes Vieira, UNICAMP, São Paulo, Brazil). After, the wells were washed 3 times with 200 µL of PBST and blocked with 200 µL of BSA 1.0% at 37 °C for 1 h. The sera of supernatants (100 µL) were added, diluted 1:2 in PBS, and the plate was incubated at 37 °C for 1 h. The wells were washed, and 100 µL of anti-human IgG AP antibody (Thermo Fisher, cat: A18820), anti-human IgA AP antibody (Thermo Fisher, cat: 31314), or anti-human IgM AP antibody (Thermo Fisher, cat: A3437), all diluted 1:2500 in PBST, were added, and the plate was incubated at 37 °C for 1 h. The antibodies bound to SARS-CoV-2 were detected with 1 mg/mL pNPP substrate (Thermo Fisher Scientific, cat: 34045) at room temperature for 30 min, and the plate was read at 405 nm on the SpectraMax M2e spectrophotometer (Molecular Devices, San Jose, CA, USA).

### 2.7. Amplification and Sequencing of Immunoglobulin Variable Domains

Total RNA from purified naive and memory B cells was extracted separately with an RNeasy mini kit (Qiagen, Hilden, Germany, cat: 74104). The cDNA was synthesized with High-Capacity cDNA Reverse Transcription Kit (Thermo Fisher, cat: 4368814), using 1 µM of the specific oligonucleotides: external Cγ CH1 (5′-GGAAGGTGTGCACGCCGCTGGTC-3′) [22], external Cα CH1 (5′-TGGGAAGTTTCTGGCGGTCACG-3′) [23] and external Cµ CH1 (5′-GGAAGGAAGTCCTGTGCGAGGC-3′) [24] for reverse transcription of IgG, IgA and IgM, respectively. The cDNA synthesis condition was 25 °C for 10 min, 37 °C for 2 h, and 85 °C for 5 min.

The heavy chain variable domains of IgG, IgA, and IgM were amplified from cDNA using the forward oligonucleotide RMX2F (5′-AAGGTGCAGCTGCTGGAGTCKGG-3′) [25] and one of the following reverse oligonucleotides: internal Cγ CH1 (5′-GTTCGGGGAAGTAGTCCTTGAC-3′) [22], internal Cα CH1 (5′-GTCCGCTTTCGCTCCAGGTCACACT-3′) [23] and internal Cµ CH1 (5′-GGGAATTCTCACAGGAGACGA-3′) [24], depending on which type of immunoglobulin. The PCR condition was 95 °C for 5 min, 30 cycles of 95 °C for 30 s, 60 °C for 30 s and 72 °C for 1 min, and 72 °C for 5 min. The amplified genetic segments were gel-extracted, purified, and sequenced by Illumina (San Diego, CA, USA) platform MiSeq 2 × 300 bp paired-end.

### 2.8. Sequence Processing

Sequences were quality-controlled using FastP v0.23.4 [26] and checked using MultiQC [27]. They were then preprocessed using the Immcantation framework [28,29]. Briefly, paired ends were assembled using Presto, keeping those with a minimum of 20 nt overlap and other parameters set to default. The forward primer was designed to pair with the framework one region of the antibody and was therefore not removed but retained for subsequent steps. The reverse primers were used as references from vaccinated individuals’ data to classify antibody types as IgA, IgG, or IgM. Duplicated sequences were collapsed to facilitate computer processing. Next, sequences were processed with the Airrflow and Nexflow automated pipeline v4.1.0 [30] using paired-end assembled sequences as the input.

Antibody annotation was conducted using IgBlastn [31]. Repertoire analyses were performed within the Immcantation framework, utilizing Alakazam v1.0.2 [28] for data pre-processing, clonal abundance estimation, and diversity analysis. The estimate Abundance method employs bootstrapping to robustly estimate clonal relative abundance distributions and confidence intervals, with normalization via uniform resampling and sample size thresholds ensuring unbiased comparisons across groups. To minimize the impact of outliers and artifacts, sequences with fewer than two duplicates were discarded, and a maximum threshold of 30% frequency for a single clone within a sample was established, as such dominance is atypical in healthy individuals and may indicate technical artifacts or expansions unrelated to baseline repertoire diversity. To ensure robust analyses, Shazam v1.0.2 [28] was used for statistical SHM models with default parameters.

Additionally, in-house developed tools were employed. As a control set, the IgG VH gene repertoire of MBC from vaccinated individuals (PCV) was compared with the IgG VH repertoire of MBC obtained from PBMC of five individuals (HD), which was collected before the COVID pandemic in 2018 and was cultivated and isolated in the same conditions. All scripts used to process antibody sequences are publicly available on GitHub v1.0.0: https://github.com/PHAB1/BioPhage (accessed on 30 March 2025).

### 2.9. Visualization of Antibody Sample Relationships Using t-SNE

We employed t-SNE (t-distributed Stochastic Neighbor Embedding) to visualize the relationships between antibody samples. This technique effectively visualizes high-dimensional data by reducing dimensions while preserving the relative distances between data points. Our analysis focused on the characteristics of the antibodies, precisely the size and similarity of the CDRH3 region. Antibodies were subsampled from each sample. To quantify similarity, we created a relationship matrix using the Levenshtein distance, which measures the minimum number of single-letter changes required to transform one sequence into another. This metric effectively captures the degree of similarity between CDRH3 sequences, enabling detailed comparisons. The resulting matrix was incorporated into the t-SNE algorithm, comprehensively visualizing antibody sample relationships based on size and sequence similarity.

### 2.10. Principal Component Analysis

Antibodies from each sample were classified according to their VDJ sequences. Before classification, raw sequence data underwent rigorous pre-processing, including quality filtering, adapter trimming, error correction, and demultiplexing to remove low-quality reads and artifacts. The sequences were then aligned to reference VDJ gene segments, and counts were standardized to correct for variations in sequencing depth and sample input. All possible classifications were quantified, and the data were normalized based on their relative frequency within each sample. Principal Component Analysis (PCA) was subsequently performed, and the R v4.4.0 programming language was used to plot the samples according to their principal components (PCs), allowing for the visualization of the major patterns and relationships in the dataset.

### 2.11. Network Analysis of CDRH3 Similarity

The r-igraph v2.0.3 package [32] was employed to analyze CDRH3 sequence similarity among antibodies within individual patients. 1000 antibodies were sampled for each patient, and connections were established between CDRH3 sequences exhibiting at least 80% identity, identical V-J usage, same length, and no gaps allowed [33]. This network-based approach enabled the identification of clusters of highly similar CDRH3 sequences within the same individual, providing a framework to investigate patterns of immune response and antibody diversity.

### 2.12. Somatic Hypermutation (SHM) Analysis

To compare somatic hypermutation (SHM) between vaccinated and pre-pandemic individuals, we employed a methodology that involved calculating the ratio of replacement (R) to silent (S) mutation frequencies based on the frequency of mutations. This approach enabled us to assess the extent of SHM by measuring the frequency of mutations that alter the amino acid class (replacement) relative to those that do not (silent). Cases where either R or S were equal to zero were excluded from the analysis to ensure meaningful comparisons, preventing undefined or infinite ratio values from affecting the results.

### 2.13. Analysis of Convergent Immune Responses

To investigate the convergent immune responses among vaccinees, RNA-seq data processing outputs and sequences from CoV-AbDab (the coronavirus antibody database) (Raybould et al., 2021) [34] were utilized. Antibody sequences were consolidated into a FASTA file, with key characteristics in the header and the CDRH3 as the sequence for comparison. Comparisons were performed in an all-against-all manner, selecting sequences that met the criteria of identical V-J gene usage, CDRH3 of the same length, and within two aa mismatches per 10 CDRH3 across different sample groups, not allowing indels or deletions. This step was essential for excluding selections from the same individual for the same IgA, IgG, or IgM class. The public database was also treated as a distinct group labeled “cov_db”.

A stringent criterion for defining convergent clonotypes was also applied to identify shared clones in the immune response to the virus among vaccinees. This required at least one antibody to be similar to another antibody group in our samples and at least one clone from CoV-AbDab.

### 2.14. Statistical Analysis and Plotting

The analysis of the expansion and proliferation of cells was measured by flow cytometry, and the unpaired *t*-test was used after checking the normality of the data. The differences were assessed using one-way ANOVA with a post hoc Tukey test in ELISA experiments. The Mann–Whitney U test was employed to compare V-family and V-gene frequencies due to its suitability for non-parametric data, which may not follow a normal distribution. Shannon diversity index was calculated for individuals PCV-03, 04, 11, and 15 across IgA, IgG, and IgM isotypes. The statistical significance of differences in the diversity index “D” between groups was assessed using a bootstrap delta distribution for each pairwise comparison. The distribution of CDRH3 lengths across IgG isotypes in vaccinated and pre-pandemic individuals was analyzed using the Mann–Whitney U test with Benjamini–Hochberg correction to control for multiple comparisons. All statistical analyses and plotting were performed using GraphPad Prism v.8.0 and R v4.4.1. The normality of the data was tested using the Kolmogorov–Smirnov test. An alpha level of 0.05 was considered for all tests, and the p-value was considered statistically significant when *p* < 0.05 *; *p* < 0.01 **; *p* < 0.001 ***.

## 3. Results

### 3.1. Expansion of Memory B Cells from Individuals Vaccinated with CoronaVac

**Figure 1:**
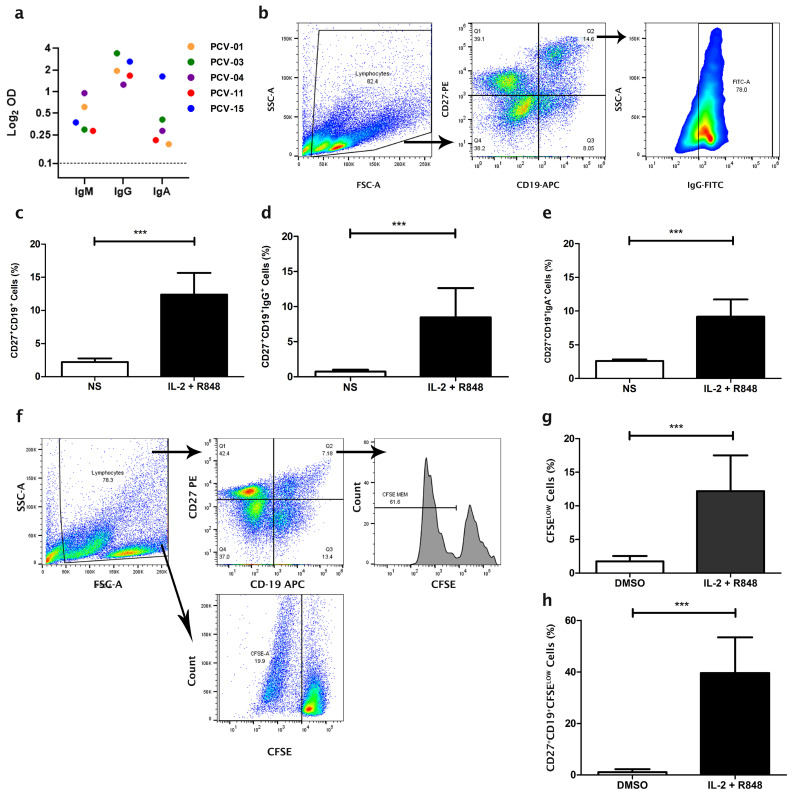
Stimulation of memory B cells (MBC) by PBMC culture with IL-2 and R848. (**a**) The presence of anti-SARS-CoV-2 IgM, IgG, and IgA in the sera from vaccinated individuals was evaluated by ELISA. The sera were collected 30 days after the second dose of CoronaVac. The dashed line represents the limit of detection of specific binding. PBMC were cultured for seven days with IL-2 and R848 or without stimuli (NS): (**b**) Gate strategy for analysis of PBMC differentiation in MBC (CD27^+^CD19^+^) and specific MBC (IgG^+^CD27^+^CD19^+^). Memory B cells are analyzed within the gate of lymphocytes, and IgG^+^ memory B cells are analyzed within the CD27^+^CD19^+^ cell gate. Percentage of (**c**) MBC, (**d**) MBC-IgG^+^, and (**e**) MBC-IgA^+^ cells concerning total PBMC. (**f**) Gate strategy for analysis of PBMC proliferation by flow cytometry. The proliferation of total PBMC (**g**) and MBC (**h**) after culturing PBMC for seven days in the presence of IL-2 (5 ηg/mL) and R848 (1 µg/mL), compared to proliferation after cultivation in the presence of DMSO (0.5%). Data are presented as Mean ± S.D. For each analysis, N = 5 individuals per group and statistical differences were identified by unpaired *t*-test, with *p* values (*p* < 0.001 ***) considered significant.

A cohort of individuals vaccinated with two doses of CoronaVac, composed of two males and three females with an average age of 34 years (SD ± 11.3), was selected for characterization (Supplementary Table S1). All selected individuals did not report having symptoms or diagnosis of COVID-19, although it is impossible to rule out the possibility of asymptomatic infection in the period before vaccination. The serology of the individuals 30 days after the second dose showed variable IgM, IgG, and IgA responses specific to SARS-CoV-2 (Figure 1a). The PCV-04 had a high reactivity for anti-SARS-CoV-2 IgM, while PCV-15 had the highest response for IgA.

PBMC from the donors were isolated and cultured in vitro to expand the number of MBCs and study the immunological repertoire of the antibody response in the context of immunization by CoronaVac. Initially, the expansion of MBC in PBMC culture was tested with different conditions of immunological impulses over various times, and the best expansion was obtained after seven days of culture with the combination of IL-2 and R848 stimuli (Supplementary Figure S1).

After culturing PBMC derived from individuals for seven days with the polyclonal stimuli of IL-2 and R848, in addition to the stimulus of the presence of T cells together, the populations of total MBC (CD27^+^CD19^+^), IgG MBC (IgG^+^CD27^+^CD19^+^), and IgA MBC (IgA^+^CD27^+^CD19^+^) increased from 2.1%, 0.71%, and 2.75% to 10.9%, 8.4%, and 8.7%, in comparison to cells without stimuli (*p* = 0.0001; *p* = 0.0032; *p* = 0.0005, respectively) (Figure 1b–e). The stimulation also improved total cell proliferation, from 1.9% to 12.1%, and the MBC proliferation, from 1.1% to 26.1%, compared to cells cultured with DMSO only (*p* = 0.0024; *p* = 0.0003, respectively) (Figure 1f–h). These stimuli did not affect cell viability (Supplementary Figure S2).

### 3.2. Expansion of the Number of Antibody-Secreting B Cells

**Figure 2:**
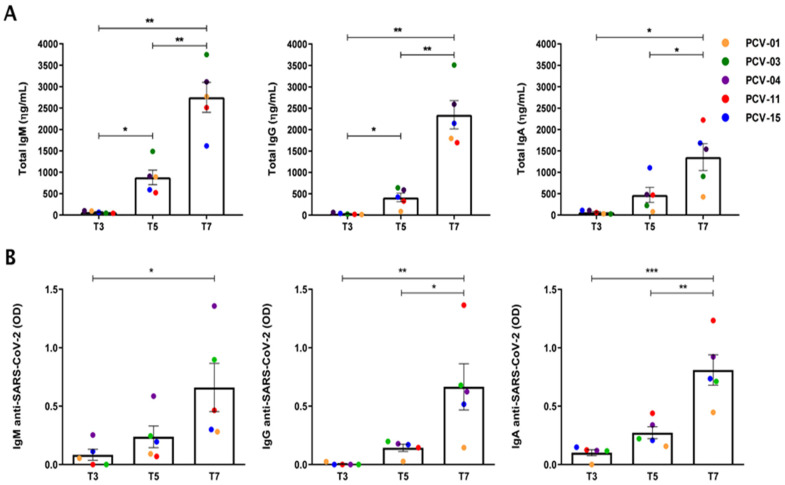
Levels of IgM, IgG, and IgA secreted in cell culture supernatants (**A**) Quantification, by ELISA, of IgM, IgG, and IgA secreted after 3 (T3), 5 (T5) and 7 (T7) days of PBMC culture with the stimuli, for each individual. (**B**) anti-SARS-CoV-2 IgM, IgG, and IgA, secreted after 3, 5, and 7 days of culture with stimuli. Each point represents Mean ± S.E.M. Statistical differences were analyzed by one-way ANOVA with post hoc Tukey test, and *p* values of *p* < 0.05 *, *p* < 0.01 **, *p* < 0.001 *** were considered significant.

Expanding the number of B cells with the stimulation protocol led to a time-dependent expansion of the antibody-producing cells, with the most secreted antibodies on the seventh day compared to days three and five (Figure 2A). On the seventh day, the individual PCV-03 exhibited a superior concentration of total secreted IgM and IgG but a smaller amount of total secreted IgA than others. The individual PCV-11 had the most significant quantitative of total IgA on the seventh day of culture. Stimulated MBC also increased the number of cells secreting antibodies specific to SARS-CoV-2 over the time of culture for all isotypes analyzed (Figure 2B). The PCV-04 had the most significant expansion of anti-SARS-CoV-2 IgM-secreting cells, while the PCV-11 showed the most significant expansion of cells secreting anti-SARS-CoV-2 IgG and IgA.

### 3.3. Antibody Patterns Differentiate Vaccinated and Pre-Pandemic Individuals

**Figure 3:**
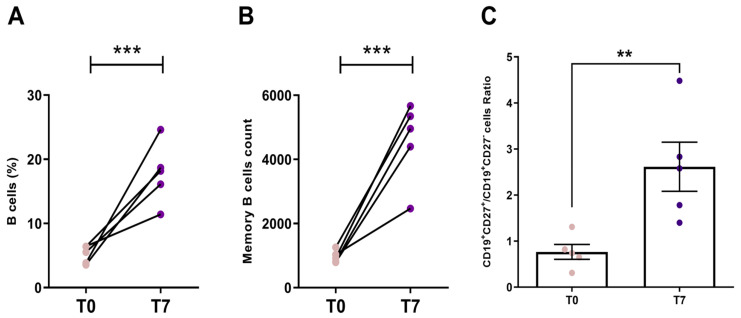
Expansion of the B cells after PBMC culture. (**A**) The difference in the B cell percentages, in relation to the PBMC population, before culture (initial time—T0, light colored dots) and after seven days of PBMC culture with the stimuli (T7, dark colored dots) (*p* = 0.0002). (**B**) Difference in memory B cell count before and after culture (*p* = 0.0001). (**C**) Ratio between CD19^+^CD27^+^ B cells and CD19^+^CD27^−^ B cells after culture (*p* = 0.0079; Data are expressed as Mean ± S.E.M). For each analysis, N = 5 individuals per group and each dot is an individual. Statistical differences were calculated by unpaired *t*-test (*p* < 0.01 **; *p* < 0.001 ***).

The percentage of B-cells in the PBMC increased significantly for all individuals after culturing with stimuli (*p* = 0.0002) (Figure 3A). The count of memory B cells was also elevated on the seventh day, compared to the condition before the culture, with an average increase of 5.4 times (Figure 3B,C). The expansion of B cells allowed their in vitro purification from the total pool of PBMC and the subsequent separation of two B cell groups: IgD^+^CD27^−^CD19^+^, corresponding to naïve cells, and IgD^−^CD27^+^CD19^+^, memory cells (Supplementary Figure S2). Both cell groups were separately sequenced, exploring IgM VH gene repertoire for naive cells and IgG and IgA VH repertoire for memory B cells (Supplementary Figure S3). The IgG MBC repertoire of vaccinated individuals (PCV) was compared to the IgG MBC repertoire of pre-pandemic individuals (HD) (Supplementary Table S1).

**Figure 4:**
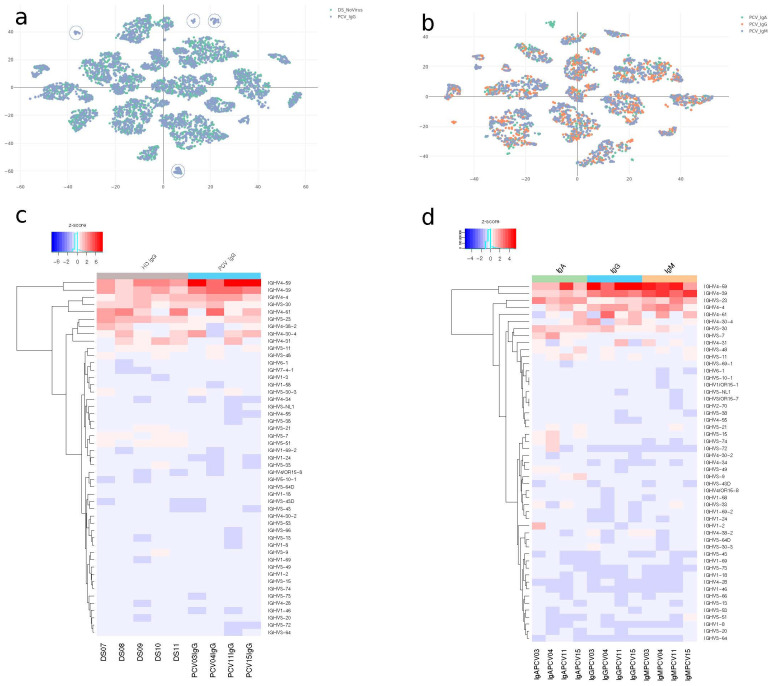
Compositional relationships across immunoglobulin isotypes. A comprehensive analysis of antibody repertoires in vaccinated individuals using multiple visualization techniques to elucidate the compositional relationships within and between different immunoglobulin isotypes. (**a**) t-SNE plots illustrating antibody repertoire clustering by CDRH3 size and identity. Comparison between vaccinated and pre-pandemic individuals, with IgG samples colored by group. Clusters containing only vaccinated individuals are circled in blue. (**b**) t-SNE plot illustrating antibody repertoire among vaccinated individuals, differentiated by immunoglobulin classes (IgA, IgG, and IgM), clustered by class. (**c**,**d**) Heatmaps representing V gene composition. (**c**) Compares V gene usage between vaccinated and pre-pandemic individuals, while (**d**) focuses on V gene composition among vaccinated individuals only.

The sequencing of donor samples resulted in 279,542 unique sequences and 52,735 distinct clones. The heterogeneity of the sequenced repertoire was explored using t-SNE dimensional reduction. Sequences were hierarchically clustered based on CDRH3 size and similarity. Repertoire from pre-pandemic individuals (HD) and repertoire from vaccinated individuals led to overlapping clusters except for small clusters of vaccinated IgG BCR sequences circled in blue (Figure 4a). The clustering also suggests a comparable heterogeneity among distinct BCR isotypes in vaccinated samples, collectively or individually (Figure 4b and Supplementary Figure S5).

PCA of the IgG BCR repertoire of vaccinated and pre-pandemic individuals showed a stronger tendency for pre-pandemic control individuals to cluster together, indicating a divergent repertoire compared to vaccinated individuals (Supplementary Figure S6a). Among vaccinated BCR isotypes, IgG and IgA compositions were diverse but eventually made clusters such as PCV-04, 11, and 15 IgA (green spheres). However, IgM BCR were more distinct and sharply biased by Principal Component 1 (PC1), which accounted for the most significant variance among samples (Supplementary Figure S6b).

This suggests distinct compositional fingerprints between the two groups. The heatmaps (Figure 4c,d) illustrate the z-score normalized expression levels of various IGHV genes across different isotypes and experimental conditions. Notably, the increased IGHV1-2 expression in the IgA PCV-03 sample (Figure 4c) is associated with clustering observed in the t-SNE and PCA (Figure 4a,b). When a specific isolated clonotype (IGHV1-2/JH3) related to a unique cluster is removed, PCV-03 IgA sample clusters with other samples in the original PCA (Supplementary Figure S7a). This specific clonotype accounts for approximately 5% of the clonal abundance in the PCV-03 individual. Similarly, in the PCV-15 patient, the most abundant clonotype related to the IGHV4-59 family also represents about 5% of clonal abundance. Removal of this clonotype also results in the PCV-15 IgG sample clustering with other IgG individuals (Supplementary Figure S7b). The t-SNE analysis revealed the antibodies causing more significant PCV-03 IgA and PCV-15 IgG divergence in PCA. The removed clonotypes clustered together and are circulated in Figure 4b and Supplementary Figure S5.

Notably, several V-genes exhibited a distinctive pattern in vaccinated individuals for the IgA isotypes (Figure 4c). However, these V-genes were not elevated in other individuals. In particular, the cited IGHV1-2 in PCV-03, IGHV3-9 in PCV-15, IGHV3-15, IGHV3-74, and IGHV3-72 in PCV-04 reflect possible specific clonal expansions in these vaccinees repertoires.

### 3.4. VH Gene Usage Adaptations After Vaccination

**Figure 5:**
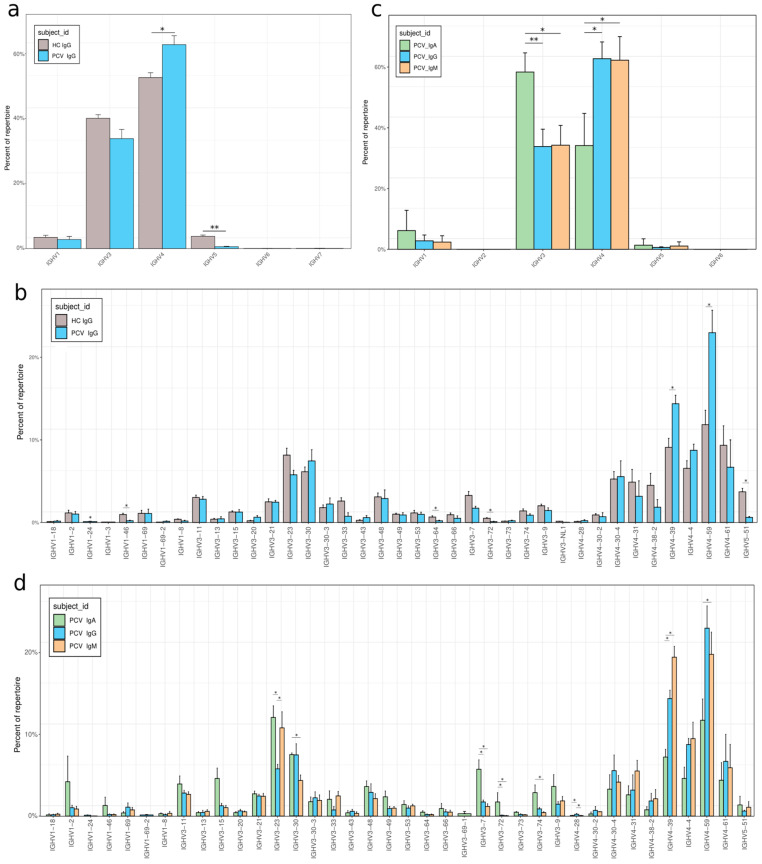
Different IGHV call distribution in response to CoronaVac vaccine. (**a**,**b**) by V gene families. (**a**) Between IgG of vaccinated and pre-pandemic individuals. (**b**) Among antibody classes IgA, IgG, or IgM. (**c**,**d**) By V gene. (**c**) Between IgG of vaccinated and pre-pandemic individuals. (**d**) Among Antibody classes IgA, IgG or IgM. Bars show mean values ± standard error of the mean. *p* < 0.05 *; *p* < 0.01 ** (Mann–Whitney U test).

To characterize the antibodies produced in response to the vaccine boost in MBC, the frequencies of IGHV genes and family usage in vaccinated individuals were compared with pre-pandemic control individuals for the IgG isotype. A significant increase in the use of the IGHV4 family and a decrease in IGHV5 were observed (*p* = 0.0476 for both) (Figure 5a). The IGHV4 increase was particularly impacted by the preferential use of IGHV4-39 and IGHV4-59 (Figure 5c). A decreased frequency of IGHV5-51 accounted for the difference in IGHV5 usage. The PCV01 sample was removed from the analysis as it exceeded the maximum threshold of 30% frequency for a single clonotype within a sample established for the samples (Supplementary Figure S8).

We further investigated the antibody classes of vaccinated individuals, analyzing and comparing the isotypes IgA, IgG, and IgM. Analyzing the IGHV families reveals a notable preference in IGHV3 gene usage associated with IgA, whereas IgG and IgM show a higher relative frequency of IGHV4 usage (*p* = 0.02857 for both) (Figure 5b). This differential usage reflects a preference of the IgA BCR for some specific VH, such as IGVH3-23, 3-30, 3-7, 3-72, and 3-74. Conversely, IgA BCR showed a reduced usage of IGHV4, such as 4-39 and 4-59.

### 3.5. Clonal Expansion and Reduced Diversity in Memory B Cells

**Figure 6:**
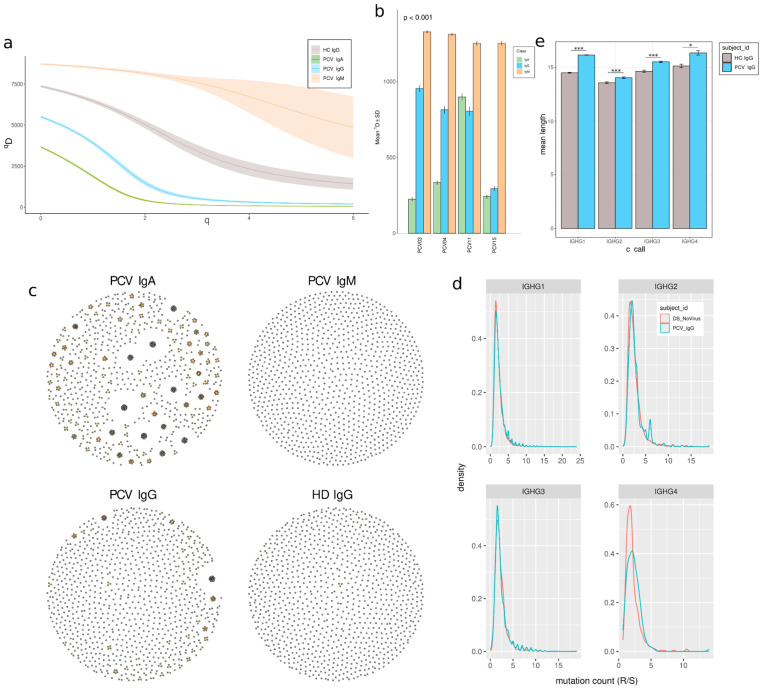
Diversity analyses and clonal network in immunoglobulin samples. (**a**) The diversity curve is calculated over a range of order q values to generate a smooth curve. This is the Hill curve where *q* = 0 indicates Richness, *q* = 1 indicates the Shannon index and *q* ≥ 2 indicates Rényi entropy. The Rényi entropy generalizes Shannon entropy to values of *q* Greater or equal 2. (**b**) Shannon diversity index for individuals PCV-03, 04, 11, and 15 across IgA, IgG, and IgM. The statistical significance of diversity index “D” differences between groups is assessed by constructing a bootstrap delta distribution for each pair of unique group values. *p* < 0.001 ***. (**c**) Igraph network plot of 1000 sampled clones from PCV-04 for IgA, IgG, and IgM, and control HC-07 for IgG. Each point represents a clone, and connections represent clonotypes (see Supplementary Figure S9 for all patients). (**d**) Distribution of mutation numbers in sequences relative to the germline, comparing somatic hypermutation (SHM) through the ratio of replacement (R) to silent (S) mutation frequencies, with patients grouped by vaccination status. (See Supplementary Figure S10 for each patient density curve). (**e**) CDRH3 Length Distribution Across IgG Isotypes in Vaccinated and pre-pandemic Individuals; Mann–Whitney U test with Benjamini–Hochberg correction. * *p* < 0.05; *** *p* < 0.001.

We analyzed clonal diversity and expansion within groups to investigate the immune response of memory B cells in vaccinated individuals. By categorizing individuals based on their antibody classes, we observed a lower diversity in the IgA and IgG isotypes in vaccinated donors compared to IgM, especially for the IgA isotype, with the lowest values of D, except for the PCV11 (Figure 6a,b). In addition, a decrease in diversity was also observed in memory IgG from vaccinated individuals compared to control memory IgG (Figure 6a). This change in diversity is accompanied by an increase in clonal expansion of antibody groups (Figure 6c), indicating increased clonal expansion of selected cells in memory cells compared to naïve IGM BCR repertoire (before class-switch), particularly for IgA. Similarly, vaccinated individuals showed increased clonal expansion compared to pre-pandemic individuals.

Clonal expansion may be accompanied by SHM, which introduces intra-clonal diversification. The vaccinated IgG BCR repertoire contains a slight increase in non-silent replacement mutations for all IgG subclasses (Figure 6d), indicating a higher mutation frequency for IgG1, IgG2, and IgG3.

The CDR3 length depends on VDJ recombination and characterizes BCR clones. CDR3 length was significantly longer in individuals who had received two doses of CoronaVac vaccination compared to pre-pandemic controls among all IgG subclasses, with the most evident shift in the IgG1 isotype (*p* = 5.484 × 10^−52^; *p* = 1.93 × 10^−5^, *p* = 1.83 × 10^−4^, *p* = 1.55 × 10^−2^, respectively, for IgG1, IgG2, IgG3, and IgG4) (Figure 6e).

### 3.6. Memory Repertoire Convergence After Vaccination

**Figure 7:**
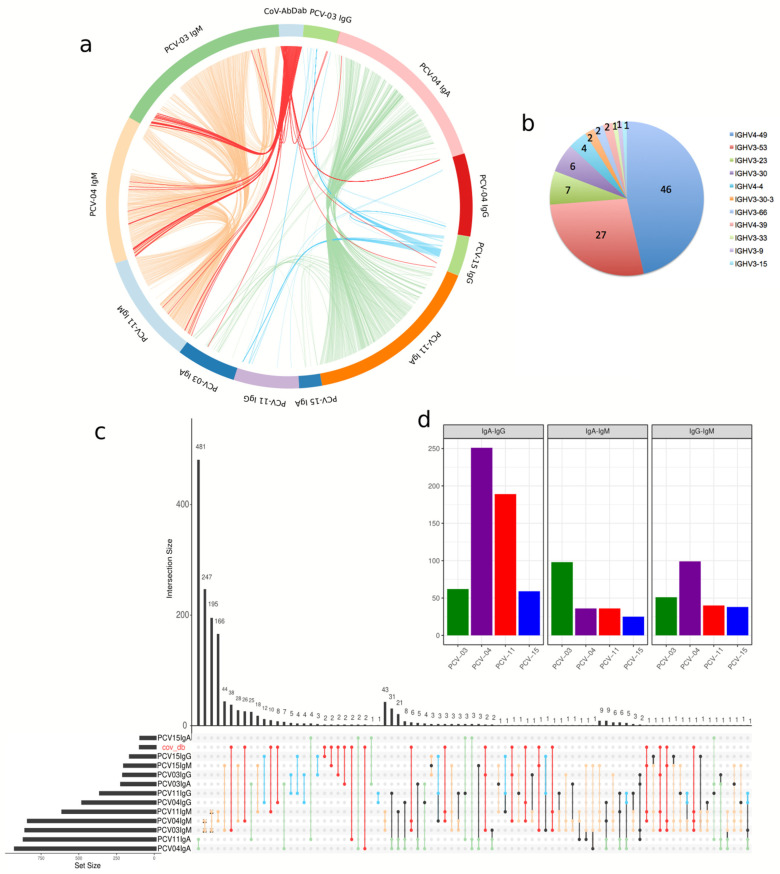
Convergent antibody responses among individuals vaccinated with CoronaVac and the CoV-abDab dataset. Similar V and J gene usage, CDRH3 length, and ≥80% sequence identity defined clonotypes. (**a**) For detecting convergent similar antibodies between vaccinated individuals (Same method as used in Table 1). The circular diagram illustrates the convergence of antibody responses. Each segment around the circle represents a vaccinated individual group or CoV-abDab antibody. The lines within the circle show the connections where the antibody responses match, with different colors representing different antibody groups. For connections, green represents IgA, cyan represents IgG, yellow-brown represents IgM, red represents any connections with CoV-abDab, and black represents other intersections. (**b**) The pie chart shows the v-gene distribution of all connections with Cov-AbDab. (**c**) The panel shows the distribution of intersection sizes of similar antibody responses among groups. The vertical bar chart represents the number of antibodies (y-axis) shared between samples. The bottom matrix highlights the specific intersections of these convergent responses. The upset plot intersections sizes are displayed in descending order of size and increasing number of intersections. Connections within the same patient or different antibody classes are not shown. For intersections, green represents IgA, cyan represents IgG, yellow-brown represents IgM, red represents any intersection with CoV-abDab, and black represents other intersections. (**d**) The bar plot represents each individual’s exclusive intersections between different antibody classes.

Focusing on the convergence of immune responses among vaccinated individuals, we explored antibody-heavy chains with similar composition among donors and isotypes to discover clones that might be vaccine-specific. Antibodies with the same VH and JH and similar HCDR3 between individuals were considered shared or convergent clonotypes and derived from similar VDJ recombination (Figure 7a,c). Our analysis detected 1447 connections among the donors’ isotypes set under this criterion (Figure 7c). Clonotypes from different isotypes from the same individual may reflect clonal expansion and class-switch events. Connections of clones containing IgA and IgG BCRs sequences were found for each vaccinated donor: PCV04 with 251 and PCV11 with 189 are among the most evident intersections, but PCV03 and PCV15 also harbor significative intersections, 62 and 59, respectively (Figure 7d).

Convergent clones were observed mainly within isotypes between pairs of donors. Interestingly, the intersection with the most connections was found between the IgA BCR sequences of donors 04 and 11, which shared 481 intersections, indicating a high degree of convergence in IgA+ MBCs between these individuals. 786 intersections were found among IgMs (non-MBC), 634 among IgA, and 41 among IgGs (Figure 7c). This result highlights a greater sharing of IgA sequences in memory B cells. Among IgG BCRs from pre-pandemic individuals, we observed no connections among them, in contrast with shared intersections among vaccinated donors.

We also examined whether these MBC-derived VHs shared similarities (see methods) with entries in CoV-AbDab (the coronavirus antibody database) (Figure 7a and Table 1). This approach identified 166 sequences from CoV-AbDab to VH sequences from vaccinees. The distribution of V-genes in these connections showed the highest occurrence in IGHV4-59, IGHV3-53, IGHV3-30-3, and IGHV3-30 (Figure 7b). Five convergent clones in IgG or IgA VHs sequences from our individuals were similar to mAbs reported to neutralize SARS-CoV-2 Wuhan original lineage and Omicron variant (Figure 7c and Table 1). We observed two IgA clonotypes from vaccinees with IGHV3-66 and IGHV4-39 that matched reported neutralizing antibodies, two IgG clonotypes with IGHV3-23 and IGHV3-33 and one IgM/IgG clonotype with IGHV4-59 (Table 1). IGHV4-39, IGHV4-59 and IGHV3-23 usage also expanded in vaccinated individuals’ repertoire (Figure 5).

## 4. Discussion

The study of antibody responses in the context of viral infections and vaccination contributes to understanding the immune mechanisms of protection against viruses and identifying antibodies with clinical relevance [15]. The investigation of the anti-virus antibody repertoire involves analysis of different populations of B cells, such as naive B cells (IgD^+^CD27^−^CD19^+^) with germline antibodies and as switched MBC (IgD^−^CD27^+^CD19^+^) with hypermutated BCR. However, the human naïve B-cells correspond to 7.6% of PBMC; human-switched MBCs and plasma cells correspond to 2–3% of B-cells and less than 0.5% of PBMC, respectively [42]; this hampers the isolation of these cell types and the fine study of the role of these cells in the immune responses. Expansion of memory B cells from ex vivo culture in the presence of immunological stimulators has been applied to overcome this difficulty and allow the study of immunoglobulin repertoire, including the case of rare clones [14,17,42,43,44].

We have previously shown that a seven-day PBMC culture protocol with IL-2 and R848 (TLR7/8 agonist) expands memory B cells, even rescuing long-term immune memory [18]. Here, we applied this protocol to expand the total B-cells without losing cell viability for individuals previously vaccinated for COVID-19. The cytokine IL-2 is known for its ability to promote the proliferation and activation of various immune cells, including B cells, crucial for vaccine studies [45]. When used along with R848, IL-2 has been demonstrated to effectively stimulate memory B cells in PBMC culture for expansion and immunoglobulin production [46]. Although studies with PBMC culture in the context of the virus show that the TLR7-directed stimulation of memory B cell proliferation appears to occur independently of T cells, the presence of T cells may still assist in the process of memory B cell expansion through cytokines and via CD40-CD40L binding. In addition, the presence of antigen-presenting cells, T cells, and B cells together mimics the immunological phenomenon in vivo [47,48].

The study of memory B cell populations through in vitro culture, such as the protocol we applied, can expand the number of clones that are initially low frequency in the cell pool of extracted PBMC, enabling the identification of rare and potentially important BCRs, difficult to assess using flow cytometry-based methods [44,49]. On the other hand, pre-culture with stimulators for B cell expansion may cause changes in the immune repertoire. However, ex vivo B cell expansion optimization studies showed that stimulation with IL-2 plus R848 or IL-21 plus CpG does not cause a bias toward a subset of immunoglobulins or induce unwanted genotypic changes [44]. In this context, we used the same culture conditions to compare the repertoire of memory B cells cultured from individuals vaccinated with CoronaVac with the pre-pandemic MBC repertoires. Additionally, we compared CoronaVac vaccinated repertoires with the SARS-CoV-2 antibody database (CoV-AbDab) to mitigate these changes and identify aspects of antibody response meaningful for SARS-CoV-2 neutralization.

After seven days of stimulated PBMC culture, MBCs were significantly expanded and there was an improvement in IgA and IgG and IgM anti-SARS-CoV-2 titers. The observed increase in specific antibody secretion activity may mainly reflect in vitro expansion derived from memory B cells. Still, a fraction of it may be derived from the differentiation of naive B cells into antibody-secreting cells, both situations in response to culture with IL-2 + R848 stimuli, in the presence of T cells, as shown in previous studies [50,51]. The increased anti-SARS-CoV-2 IgM-secreting activity in the culture supernatant from individual PCV-04 paralleled its highest anti-SARS-CoV-2 IgM response in the serum. At the same time, the anti-SARS-CoV-2 IgG-secreting activity from PCV-03 also correlated with its specific IgG reactivity in the serum, revealing that the cell expansion of the culture tended to follow the response pattern of the antibody repertoire of these individuals. Surprisingly, although the individual PCV-11 had a low signal of anti-SARS-CoV-2 antibodies in serum and a small amount of total IgG in the post-culture supernatant, this individual showed the highest signal of IgG and IgA anti-SARS-CoV-2 after the stimulus culture; thus, the individual PCV-11 differed from the behavior of the other individuals. This difference may have been caused by the fact that PCV-11 MBC harbors high-affinity virus-specific IgG and IgA. Hence, although antibody titers were lower in the pool of serum antibodies, after culture, these potential high-affinity clones expanded, resulting in a greater anti-SARS-CoV-2 titer in the supernatant. These findings reinforce the idea that the MBC expansion protocol allows the increase in the number of rare MBC clones in peripheral blood, making it possible to enrich these cells for high-performance sequencing studies [15,16]. Moreover, MBC expansion enables the rescue of already expanded clones and other clones induced by vaccination [43]. Some of these high-affinity clones could be lost in different immune cell isolation methodologies that do not involve a prior MBC number enrichment step [16].

We analyzed four individuals who received two doses of CoronaVac. Sample PCV-01 was removed as it showed a dominating clonotype that accounted for ~60% of the sample. Analysis of the BCR repertoires from individuals by sequencing revealed that the vaccinated individuals have a distinguishable repertoire, differing in IgG CDRH3 length and VH gene usage compared to a pre-pandemic repertoire. The observed elongation of CDRH3 in IgG aligns with prior studies demonstrating that more extended CDRH3 regions enhance antibody–antigen affinity and specificity [52]. Furthermore, CoronaVac was shown to promote the expansion of specific MBC clones, observed as a reduction in the IgG and the IgA BCR diversity, with a V gene usage favorable for anti-SARS-CoV-2 activity. This interpretation is coherent with the observation of an improvement in the IgG and IgA anti-virus titers during MBC stimulation. The diversity of the IgA BCR repertoire was shallow and dominated by fewer clones. Furthermore, the switched isotypes BCR repertoires of vaccinees were less diverse than the naïve IgM BCR repertoire, evidencing differences between switched memory B cells and Naive B cells in the context of this vaccine.

Clonal expansion and SHM impact gene usage, and VH gene families observed expanded in MBC of immunized individuals may reflect the effect of the vaccination. Considering the limitation of this study with a limited number of analyzed subjects, consistent differences among donors’ MBC could be accounted for the vaccination-dependent memory. As observed here in the MBC of vaccinated subjects, an increase in IGHV4 frequency and a reduction in IGHV3 and IGHV5 have also been found in COVID-19 convalescents in comparison to healthy individuals [53], with greater use of IGHV4-4, IGHV4-30-4, and IGHV4-39. Despite not observing differences in the first two V genes, we observed that IGHV4-39 and IGHV4-59 expanded in IgM and IgG repertoires and were among the most VH enriched in vaccinees compared with pre-pandemic control individuals. Both V genes have previously been found in some neutralizing antibodies, which have been shown to potent neutralize against different SARS-CoV-2 variants [54,55].

The use of the IGHV3 family is reduced in the repertoires of immunized individuals, but it still dominates the set. Clones harboring the IGHV3-30 V gene segment were expanded in the IgG and IgA BCR repertoires compared to IgM. Antibodies using IGHV3-30 have been associated with convergent antibody response to Subunit S2 of Spike from SARS-CoV-2 [56]. Among IGHV3, the IGHV3-9 gene segment was found to expand in the IgA repertoire of PCV-11 and PCV-15. This V gene was associated with the response after vaccination with the mRNA-1273, and some antibodies with IGHV3-9 have exhibited neutralizing activity against different variants of SARS-CoV-2, such as Beta, Delta, Omicron BA.1, and Omicron BA.2.75 [57]. The preferential use of IGHG3 and IGHG4 subfamilies has been widely documented in individuals receiving inactivated vaccines such as BBIBP-CorV [40]. However, our study identified distinct subfamily biases that could reflect genetic or region-specific influences on B-cell receptor (BCR) repertoire composition but could also stem from variations between BBIBP-CorV and CoronaVac despite both being inactivated platforms.

IGHV1-24, a sparsely used V gene but significantly augmented in our vaccinated cohort, has also been increasingly observed in COVID-19 convalescents and immunized individuals. Its germline form is related to antibodies to the N-terminal domains of the SARS-CoV-2 spike protein [58]. Interestingly, some neutralizing antibodies carrying IGHV1-24 against non-RBD regions were identified as resistant to mutations in SARS-CoV-2 variants [58,59].

Shared clonotypes, defined as meta-clonotypes, may represent convergent development of clones with quasi-identical VDJ recombination in more than one individual [33]. We identified convergent antibody responses in the different vaccinated individuals, mainly for individuals PCV-04 and PCV-11 who showed greater production of anti-SARS-CoV-2 antibodies during MBC expansion, and mainly for IgA response, which emphasizes the idea of the importance of IgA antibodies, not only in the mucosa but also in the serum, for the neutralization of SARS-CoV-2 [60]. Serum IgA against Spike protein and non-Spike antigens, similar to our CoronaVac antigenic context, have been dominant in the initial neutralizing antibody response [9,61]. Furthermore, the sharing of VH sequences between IgG and IgA, as we observed in the repertoire of vaccinated individuals, has also been reported in the context of viral immunizations [60,61,62].

Convergent antibodies, with recurrent VDJ recombination in different individuals, have been shown to lead to potent neutralizing antibodies to SARS-CoV-2 and may have clinical relevance [63]. We found that the sequences of some convergent IgG and IgA antibodies in our cohort carrying IGHV4-39 and IGHV4-59 families, which also dominated the V gene repertoire in response to CoronaVac, were similar to previously characterized monoclonal antibodies with the ability to neutralize the Omicron lineage [38,41]. Thus, convergent antibodies, boosted by CoronaVac in analyzed individuals, may have neutralizing capacity against SARS-CoV-2 lineages, including subsequent Omicron sublineages. In addition, these convergent antibodies may also neutralize against other coronaviruses, such as HKU1 and OC43, given the possibility that individuals had previous contact with other common coronaviruses before the study.

This study elucidates the effect of CoronaVac on the immune response, contributing to the understanding of the neutralizing action of antibodies for SARS-CoV-2 infection. Considering that CoronaVac is a whole virus vaccine and that there are still few studies of the immunoglobulin repertoire in this vaccine context compared to other vaccines, more in-depth evaluations of the impact of CoronaVac may contribute to future therapies and to the improvement of vaccination approaches. The use of the CoronaVac vaccine seems to show a protective effect on vaccinated communities [64]. Still, concerns about its effectiveness arose when novel SARS-CoV-2 variants started to substitute the initial Wuhan virus [4], mainly with the emergence of the Omicron variant compared to other vaccines [65].

However, applying a third dose of CoronaVac seems to boost immunity to SARS-CoV-2, including the Omicron lineage [65,66]. Wang et al. (2022), studying MBC repertoires from individuals who received two or three doses of the CoronaVac vaccine, identified potent neutralizing antibodies against different SARS-CoV-2 variants, including Omicron, which protect challenged mice [67]. Li et al. (2021) identified antibody sequences derived from BCRs that could be employed in treatments for patients who had recently recovered from an early-stage infection with the SARS-CoV-2 virus [68]. Our observations of VH repertoire from MBC corroborated the suggestion that CoronaVac may be protective for preventing COVID-19. However, our results do not represent direct functional analyses of the efficacy of this vaccine. Moreover, these findings highlight the significance of repertoire analysis in discovering antibodies that can be potentially neutralizing, and they serve as a crucial guide for advancing effective vaccines and therapies against evolving variants of SARS-CoV-2.

Thus, through the ex vivo expansion approach of naïve and memory B cells followed by sequencing, we identified significant IgG and IgA antibody responses to CoronaVac stimulation, especially from the IGHV3 and IGHV4 families. Considering the difficulty in determining whether vaccinated individuals had contact with SARS-CoV-2 naturally, before or during the vaccination period, we used the immune repertoire before the COVID-19 pandemic as a control. A limitation of this study is the sample size, with few vaccinated individuals and few pre-pandemic individuals being analyzed. However, previous repertoire studies using few individuals evaluated essential responses in the context of vaccines and infections for SARS-CoV-2 through high-throughput sequencing [69,70]. The comparison between expanded antibodies in vaccinated individuals and neutralizing antibodies from the CoV-AbDab database strengthens the evidence of changes in the BCR repertoire found after immunization with CoronaVac. Yet, we did not perform any direct neutralization assay with the antibodies we identified. Future longitudinal studies with these individuals after the third dose booster, as well as analyses characterizing the neutralizing activity of IgGs and IgAs identified in the repertoire of these individuals, may deepen the understanding of the effectiveness of CoronaVac.

## 5. Conclusions

We demonstrated that the CoronaVac whole virus vaccine causes changes in the humoral immune repertoire by expanding antibody clones IGHV4-39 and IGHV4-59 and altering the CDRH3 patterns. We also showed differences in the responses of naïve B cells and memory B cells regarding IgM, IgG, and IgA after vaccination. With a protocol to expand the number of circulating B cells combined with deep sequencing analyses, we were able to identify convergent clones among vaccinated individuals and neutralizing antibody database. In addition, we identified meaningful anti-SARS-CoV-2 immune responses, such as some IgA antibodies, suggesting that CoronaVac can mount a protective humoral memory yielding antibodies potentially virus neutralizing.

## Figures and Tables

**Table 1:** COVID-19 clonotypes with identical V-J and CDRH3 regions between individuals and CoV-AbDab.

Name	Binds	Neutralize	Epitope	Subject ID	C Call	V Call	J Call	CDRH3 *	Reference
BD55-1970 10D12 Ab_56D7	CoV2	yes	RBD	PCV-04	IgA	IGHV3-66	IGHJ6	ARDLDYYGMDV (×3) ARRLDYYVMDV	[35,36,37]
AZ262	CoV2	yes	RBD	PCV-03	IgA	IGHV4-39	IGHJ6	ARLTRGYSYGYSMDV ARDGRGYSYGYGMDV	[38]
WCSL-36	CoV2	yes	RBD	PCV-15	IgG	IGHV3-23	IGHJ5	AKSR Q L A FDP AKSRDLVFDP	[39]
Wang-C443	CoV2	yes	RBD	PCV-03	IgG	IGHV3-33	IGHJ4	ARE D YYDSSG S LDY ARENYYDSSGYLDY	[40]
C1414	CoV2	yes	RBD	PCV-04 PCV-03	IgM IgG	IGHV4-59	IGHJ5	ARHYDSSGYTYNWFDP ARTYDSSGYYPNWFDP ARTYDTSGYYANWFDP **	[41]

* The sequence in green is the reference sequence from a vaccinated individual compared to the others. Above the reference sequence, we have the sequences obtained from CoV-AbDab, and **. The orange sequence represents a non-reference alignment from a vaccinated individual. Amino acids that are substituted are highlighted in red.

## Data Availability

The raw sequencing data from samples are available in the NCBI Sequence Read Archive (SRA) under BioProject accession number PRJNA1154681.

## Supplementary Materials

The following supporting information can be downloaded at: https://www.mdpi.com/article/10.3390/vaccines13040393/s1, Table S1. Characteristics of the individuals. Figure S1. (a) Evaluation of the CD27+CD19+ B cell percentage (in relation to the total PBMC) in three, five and seven days of PBMC culture with different stimuli. (b) Proliferation of memory B cells in different days of PBMC culture. UNS: unstimulated cells. IL-2: 5 ng/mL. R848: 1 µg/mL. Figure S2. Analysis of cell viability after PBMC culture for seven days with IL-2 + R848 stimuli. Figure S3. After the PBMC culture with stimuli, the populations of naïve B cells (CD27−CD19+IgD+) (a) and memory B cells (CD19+CD27+IgD−) (b) were purified and the total RNAs from both type of cells were extracted separately. Figure S4. Workflow overview for pre-processing and analysis of NGS antibody repertoires. Figure S5. t-SNE plot illustrating antibody repertoire among vaccinated individuals, differentiated by immunoglobulin classes (IgA, IgG, and IgM); clustered by patient and class. Figure S6. (a and b) Principal Component Analysis (PCA) using V-D-J composition similarities among samples. Figure S7. The removal of specific abundant clonotypes results in the affected samples clustering more closely with other samples in the PCA analysis. Figure S8. Comparative analysis of PCV-01 clonal abundance and network structure across immunoglobulin classes. Figure S9. Igraph network plot of IgM, IgG, and IgA from 4 vaccinated patients and IgG from 5 pre-COVID-19 pandemic individuals. Nodes represent clones, and connections represent clonotypes. Figure S10. Density curves showing the ratio of replacement (R) to silent (S) mutation frequencies for IGHG1, IGHG2, IGHG3, and IGHG4 in each patient.
