# Supplementary material for: Naive and Memory B Cell BCR Repertoires in Individuals Immunized with an Inactivated SARS-CoV-2 Vaccine
Source: Vaccines (Basel). 2025 Apr 8;13(4):393. doi: 10.3390/vaccines13040393 (PMC12031002; doi:10.3390/vaccines13040393)
Supplement: Supplementary file 1 [file vaccines-13-00393-s001.zip › vaccines-3522767-supplementary.pdf]

Supplementary Table S1. Characteristics of the individuals

INDIVIDUAL	Age (years)	Sex	Dose I	Dose II
PCV-01	35	F	03/23/21	04/21/21
PCV-03	28	M	01/27/21	02/24/21
PCV-04	30	F	03/31/21	04/28/21
PCV-11	53	F	03/28/21	04/25/21
PCV-15	24	M	08/21/21	09/18/21
**Mean**	**34**			
HD-01	27	F	-	-
HD-02	29	F	-	-
HD-03	28	F	-	-
HD-04	30	M	-	-
HD-05	38	F	-	-
**Mean**	**30**			

PCV: Individual vaccinated with CoronaVac. HD: control individual whose PBMC was collected in 2018, before the COVID pandemic.

Abbreviations: F (female), M (male). Gender defined by self-report.

None of the individuals reported having had diagnosis or symptoms of COVID-19. None of the individuals have comorbidities.

Blood samples were collected from individuals 30 days after the second dose.

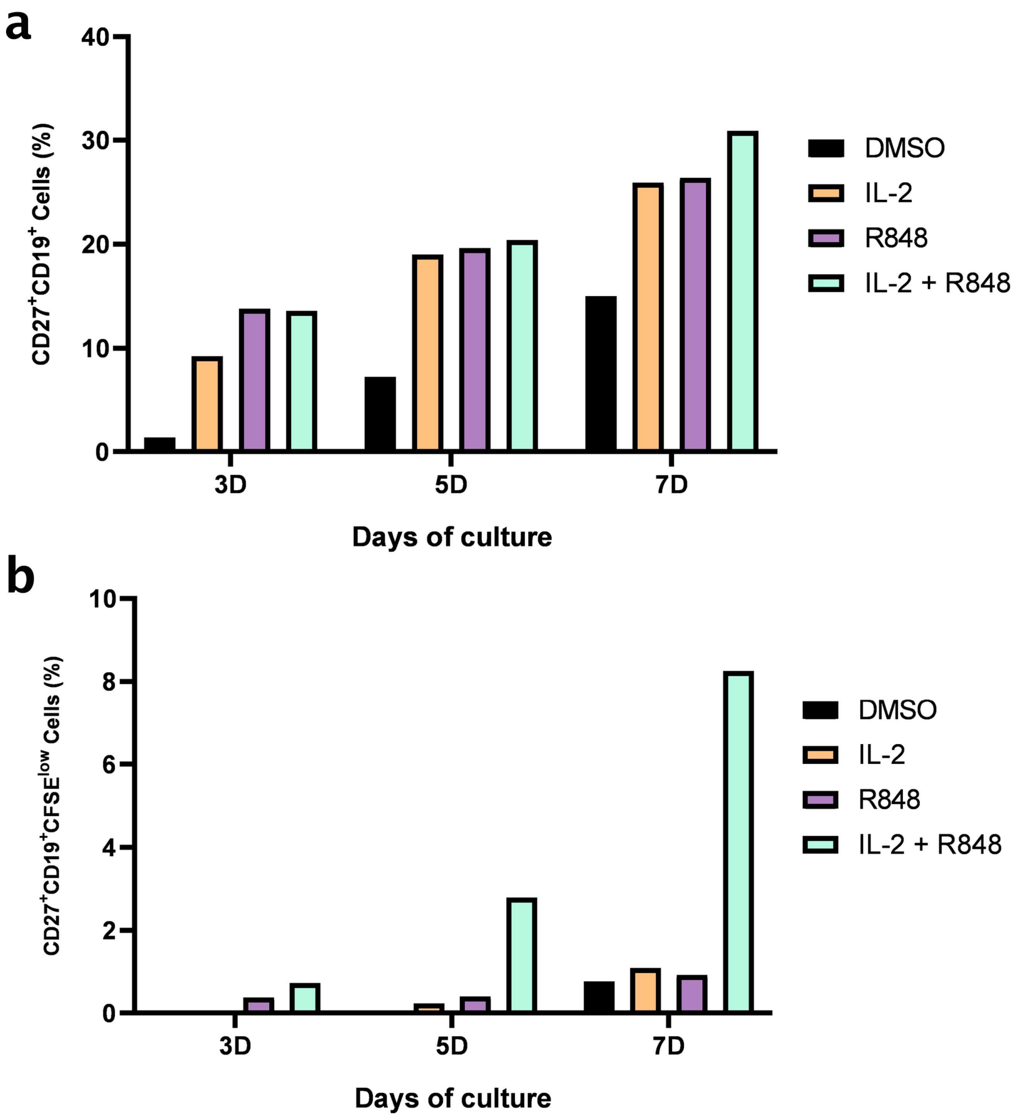

Supplementary Figure S1. (**a**) Evaluation of the CD27+CD19+ B cell percentage (in relation to the total PBMC) in three, five and seven days of PBMC culture with different stimuli. (**b**) Proliferation of memory B cells in different days of PBMC culture. UNS: unstimulated cells. IL-2: 5 ng/mL. R848: 1 ug/mL.

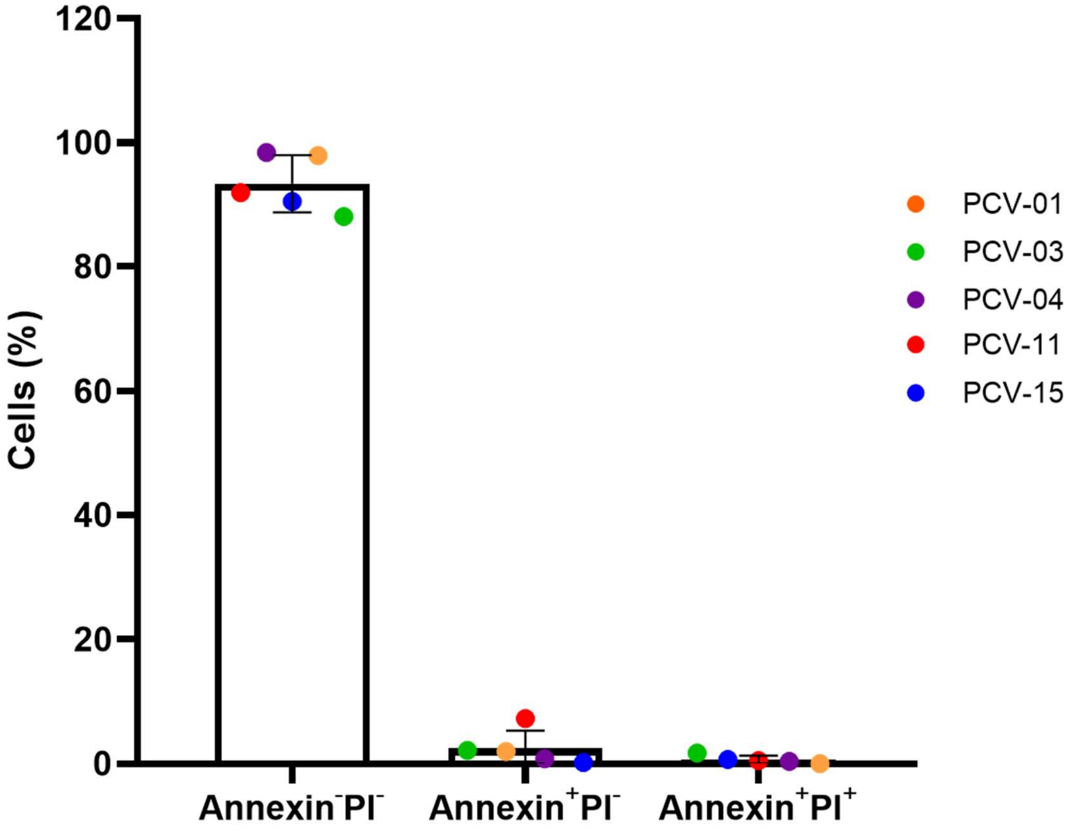

Supplementary Figure S2. Analysis of cell viability after PBMC culture for seven days with IL-2 + R848 stimuli. Viable cells (Annexin-$V^{-}PI^{-}$) were quantified by flow cytometry and distinguished from cells in early apoptosis (Annexin-$V^{+}PI^{-}$) and cells in late apoptosis (Annexin-$V^{+}PI^{+}$). Annexin-V was measured with APC staining. All percentages were calculated based on the total number of cells acquired (ungated).

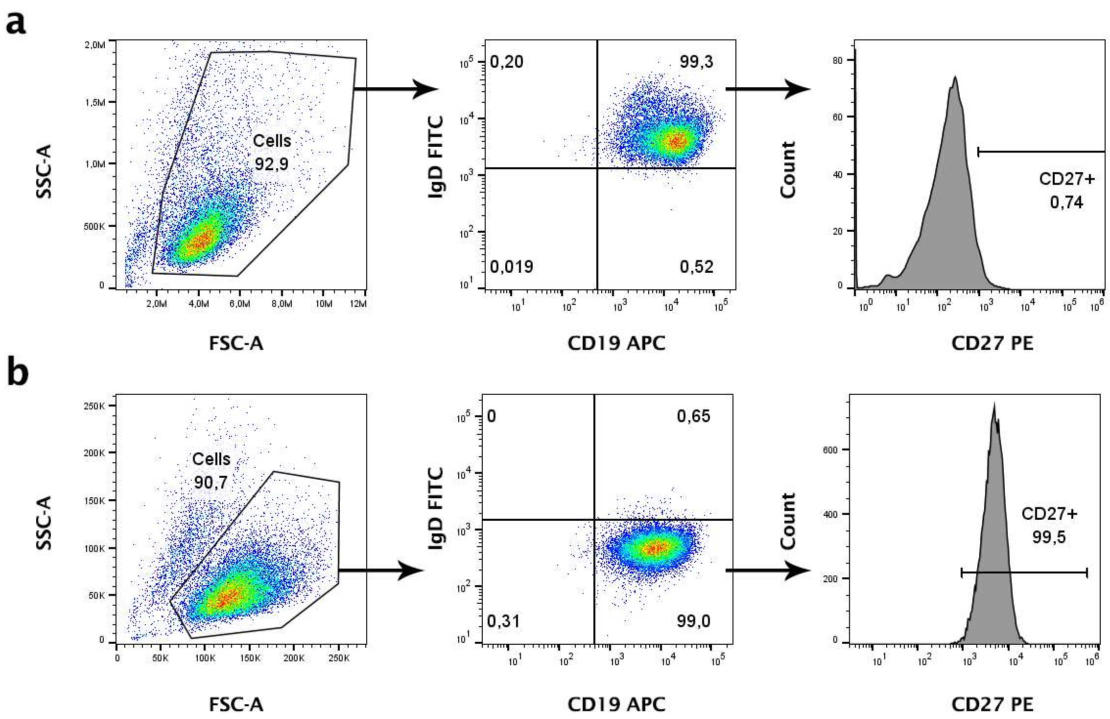

Supplementary Figure S3. After the PBMC culture with stimuli, the populations of naïve B cells (CD27-CD19+IgD+) (**a**) and memory B cells (CD19+CD27+IgD-) (**b**) were purified and the total RNAs from both type of cells were extracted separately. Representative data from individual PCV-04.

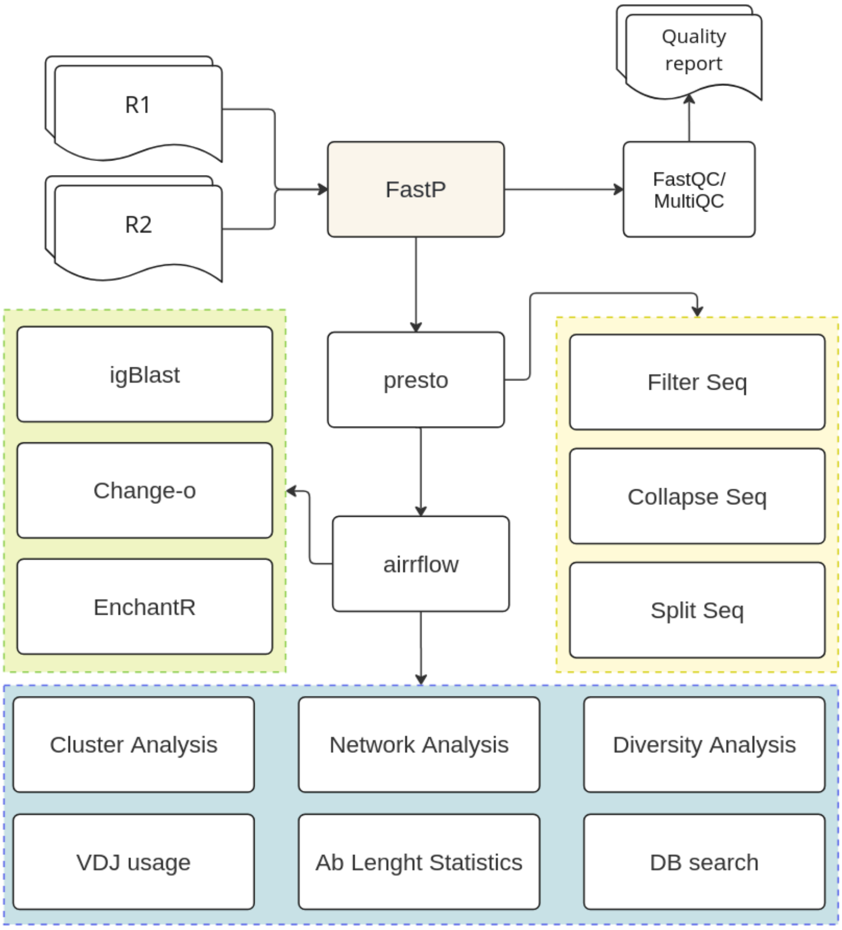

Supplementary Figure S4. Workflow overview for pre-processing and analysis of NGS antibody repertoires.

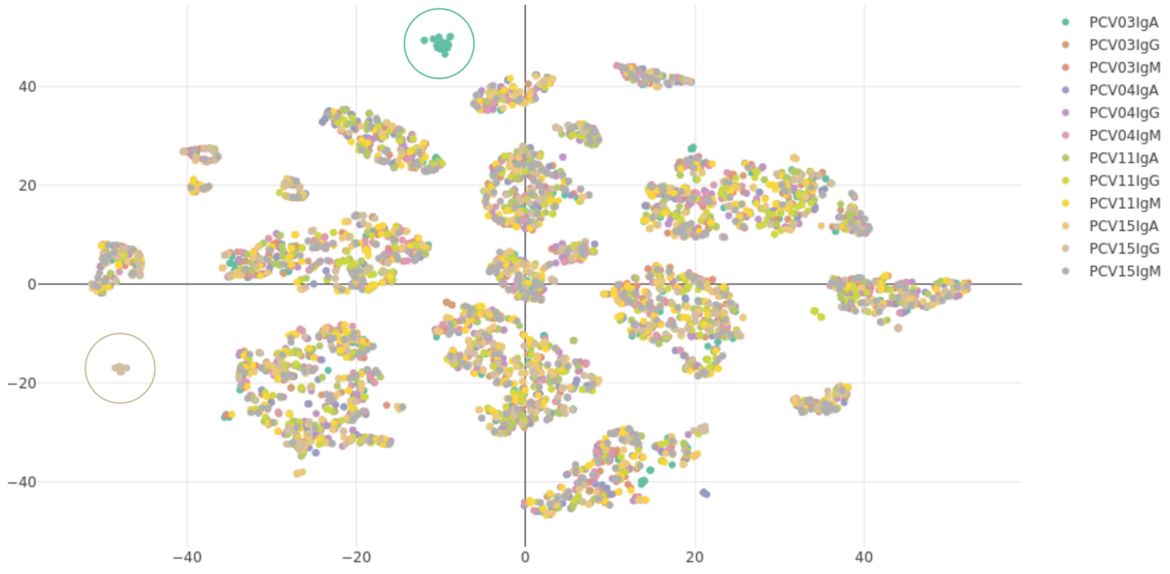

Supplementary Figure S5. t-SNE plot illustrating antibody repertoire among vaccinated individuals, differentiated by immunoglobulin classes (IgA, IgG, and IgM); clustered by patient and class. Clusters containing clonotypes of a single isotype and belonging to an individual were circled in cyan and yellow-brown for PCV-03 IgA and PCV-15 IgG, respectively.

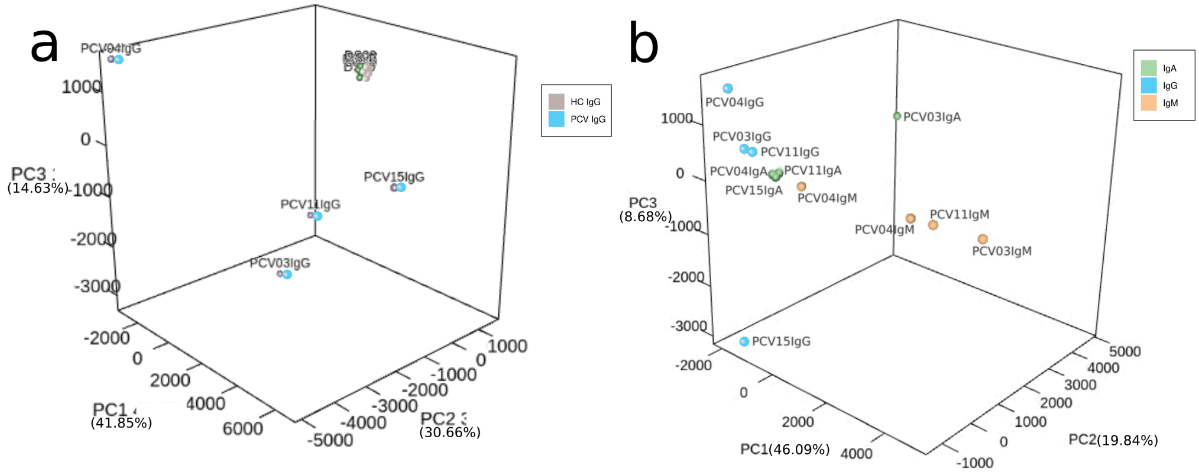

Supplementary Figure S6. (**a** and **b**) Principal Component Analysis (PCA) using V-D-J composition similarities among samples. **(a)** compares antibody repertoires between vaccinated and pre-pandemic individuals. (**b**) focuses on vaccinated individuals, with clusters colored by antibody class.

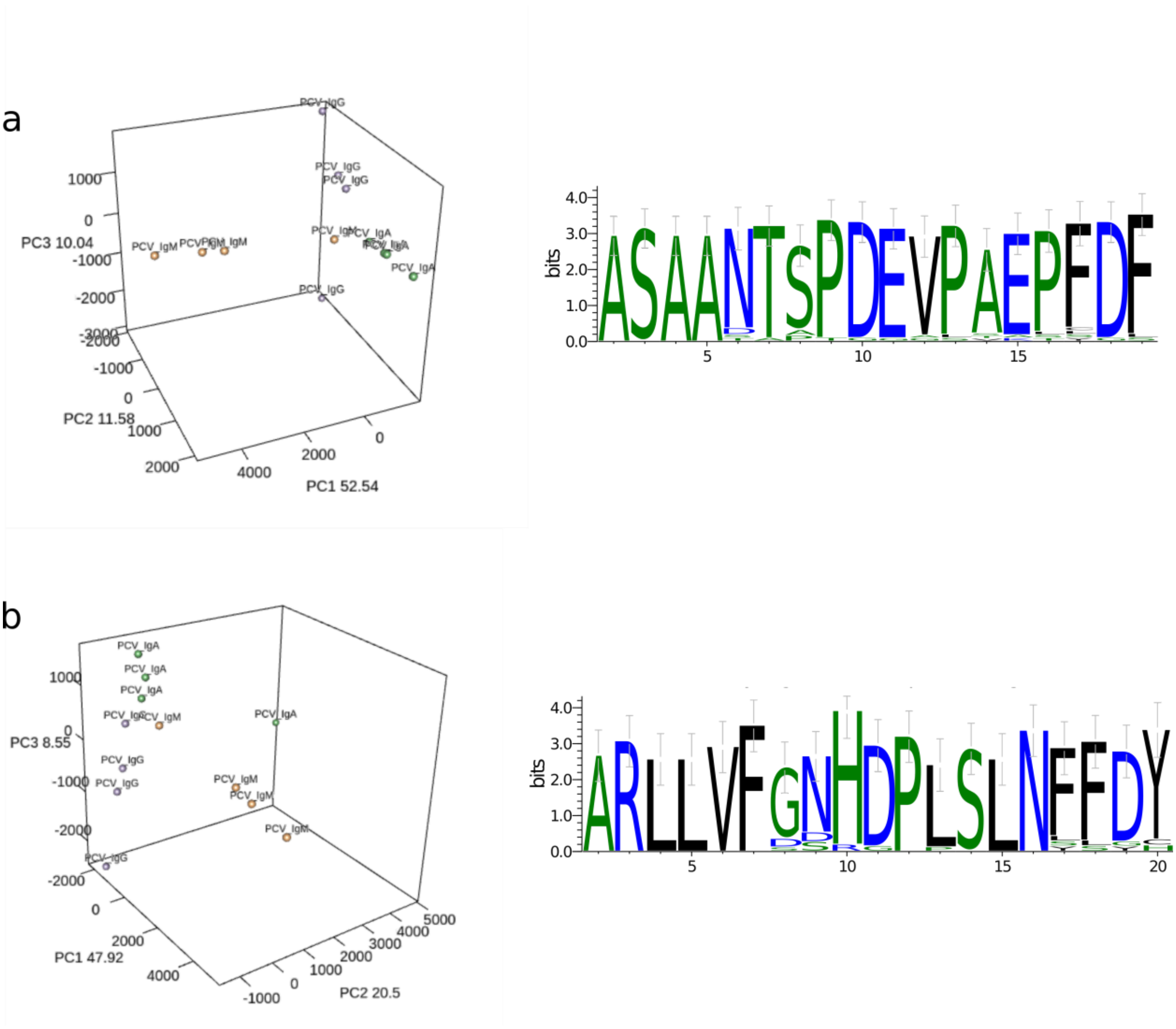

Supplementary Figure S7. The removal of specific abundant clonotypes results in the affected samples clustering more closely with other samples in the PCA analysis. (a) Removing the specific isolated clonotype (IGHV1-2-JH) related to a unique t-sne cluster causes the PCV- 03 IgA to cluster more closely with other individuals IgA. (b) Removing the most abundant clonotype related to the IGHV4-59 family in the PCV-15 patient causes the PCV-15 IgG to cluster more closely with other individuals IgG.

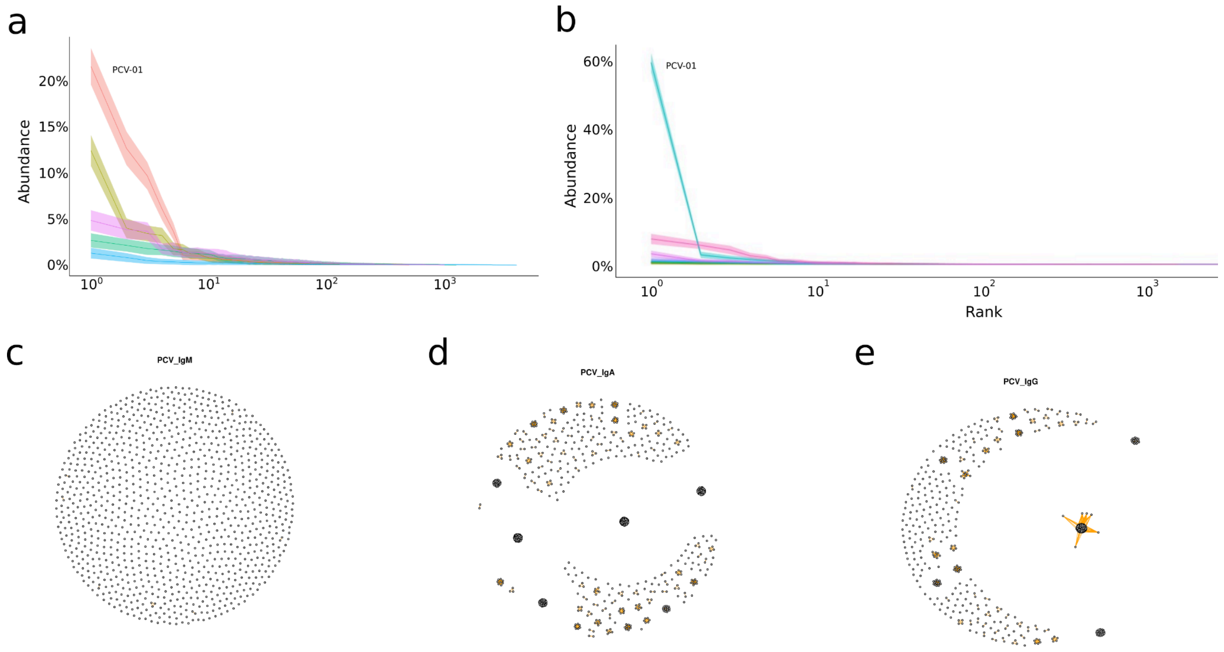

Supplementary Figure S8. Comparative analysis of PCV-01 clonal abundance and network structure across immunoglobulin classes. (a, b) Rank-based clonal abundance distributions for IgA (a) and IgG (b), both highlighting a disproportionately high abundance of PCV-01. (c, d, e) iGraph netplots of PCV-01 for IgM (c), IgA (d), and IgG (e). Nodes represent clones, and connections represent clonotypes.

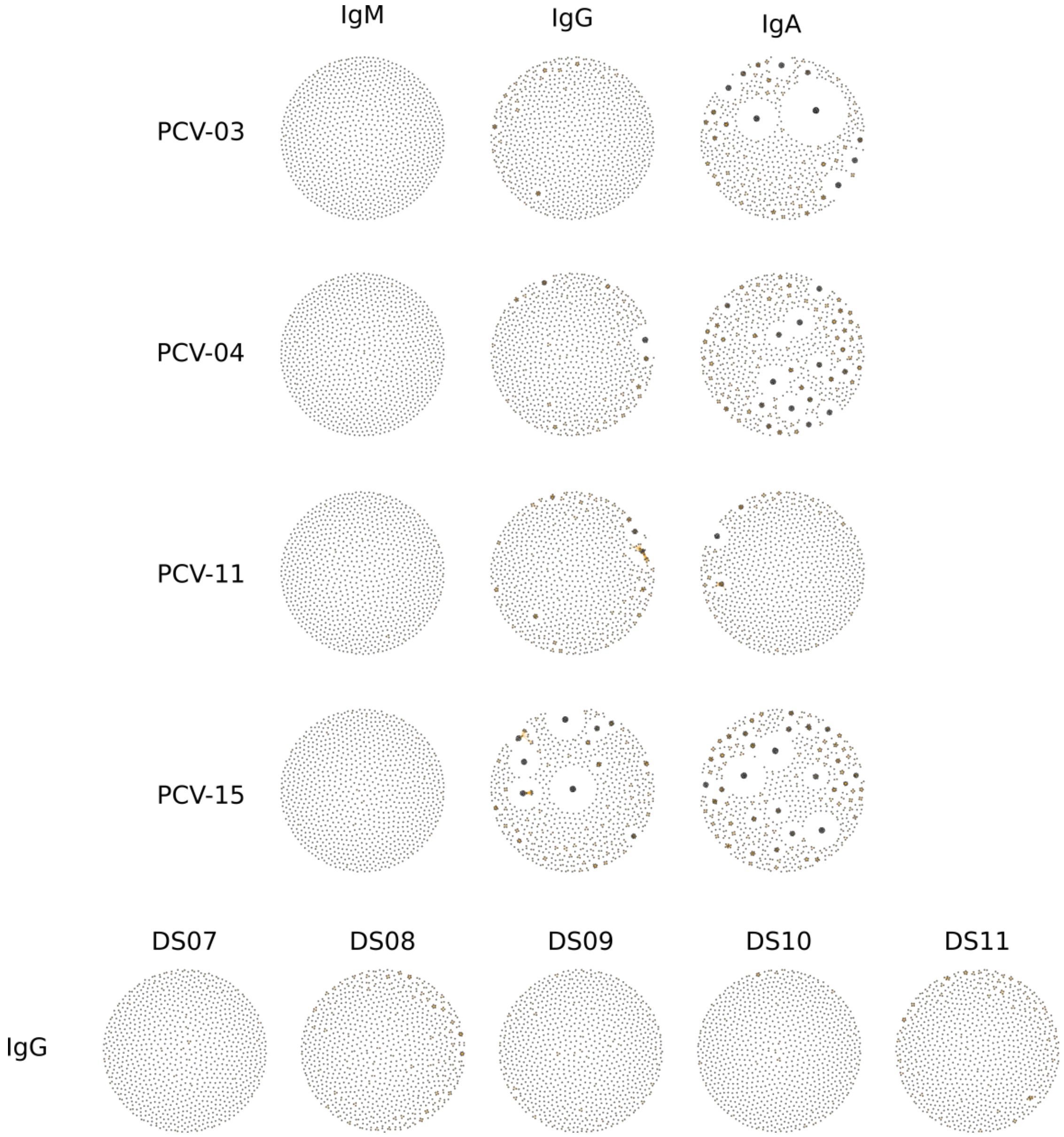

Supplementary Figure S9. Igraph network plot of IgM, IgG, and IgA from 4 vaccinated patients and IgG from 5 pre-COVID-19 pandemic individuals. Nodes represent clones, and connections represent clonotypes.

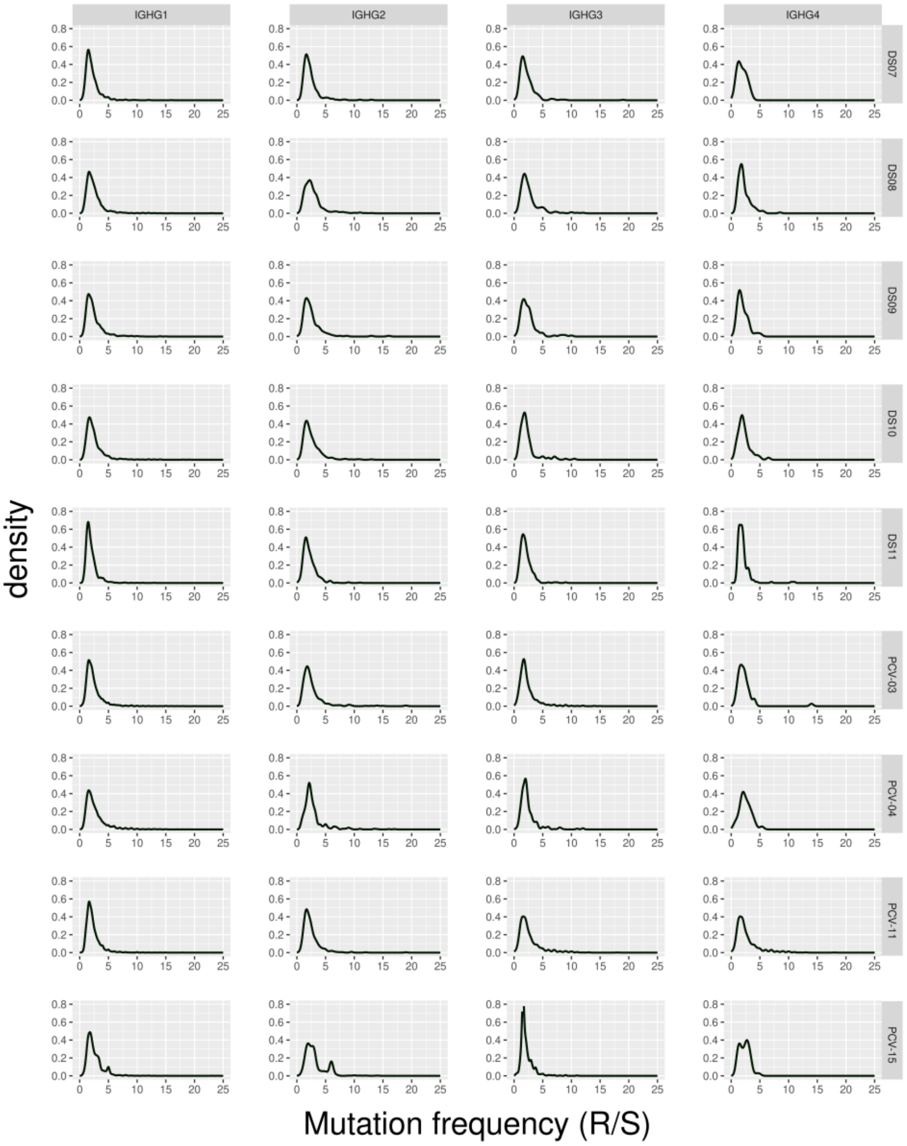

Supplementary Figure S10. Density curves showing the ratio of replacement (R) to silent (S) mutation frequencies for IGHG1, IGHG2, IGHG3, and IGHG4 in each patient.
